
JOURNAL INFORMATION
==============================
Journal ID: Wirtschaftsdienst

Wirtschaftsdienst

EISSN: 1613-978X

ARTICLE INFORMATION
==============================
PMCID: PMC9466312
DOI: 10.1007/s10273-022-3265-6
Article ID: 3265
Article version: 1
Subjects: Ökonomische Trends

Ausländische Fachkräfte im Blickfeld

Focus on foreign skilled workers

Haustein Erik haustein@hwwi.org

grid.435054.3 0000 0001 2227 8589 Hamburgisches WeltWirtschaftsInstitut gemeinnützige GmbH (HWWI), Oberhafenstraße 1, 20097 Hamburg, Deutschland
Electronic publication date: 2022 Sep 12
Print publication date: 2022
Volume: 102
Issue: 8
First page: 655
Last page: 656
Copyright: © Der/die Autor:in 2022
License: Open Access: Dieser Artikel wird unter der Creative Commons Namensnennung 4.0 International Lizenz veröffentlicht (https://creativecommons.org/licenses/by/4.0/deed.de).
Open Access wird durch die ZBW — Leibniz-Informationszentrum Wirtschaft gefördert.
License URL: https://creativecommons.org/licenses/by/4.0/

Keywords: J21, J23, J61
issue-copyright-statement: © The Author(s) 2022

==============================

Nach mehr als zwei Jahren COVID-19-Pandemie und dem Beginn der Sommerferien treibt die wiederaufkommende Reiselust die Deutschen in weit entfernte Länder. Allerdings ist eine Flugreise derzeit von langen Warteschlangen bei den Sicherheitskontrollen und Problemen in der Gepäckabfertigung gekennzeichnet. Beides stellt die Geduld der Urlauber:innen auf die Probe. Und obwohl in einigen Bundesländern die Ferienzeit bereits zur Hälfte verstrichen ist, scheint in den nächsten Wochen keine Besserung in Sicht zu sein. Der wiedererstarkende Tourismus zeigt unverblümt ein strukturelles Problem des Flugverkehrssektors auf, das exemplarisch für die ökonomische Situation Deutschlands steht: ein akuter Fachkräftemangel.

Dem waren erhebliche, in weiten Teilen unfreiwillige berufliche Abwanderungen von sowohl Technik- als auch Servicefachkräften der Luftfahrtbranche während der Coronapandemie 2020/2021 vorausgegangen. Diese fanden attraktive Anstellungen z.B. in den Bereichen der Logistik oder des Online-Handels (Burstedde und Koneberg, 2022). Da die Rückgewinnung der abgewanderten Fachkräfte mit Schwierigkeiten verbunden ist, soll die Anwerbung ausländischer Fachkräfte den personellen Engpass kurz- bis mittelfristig reduzieren. Einwanderung wird hierbei nicht nur als unmittelbare Lösung verstanden, sondern soll auch einer Verschärfung der Situation durch den demografischen Wandel präventiv entgegenwirken. Jüngst stellte der Deutsche Industrie- und Handelskammertag ein Maßnahmenpaket vor, das den Zugang zum Arbeitsmarkt vereinfachen und der Fachkräfteverknappung entgegenwirken soll (DIHK, 2022).

Die politischen Forderungen und der ökonomische Bedarf stehen dabei im starken Kontrast zu den Entwicklungen der Einwanderungszahlen der letzten Jahre. Bedingt durch die Pandemie und die damit verbundenen Eindämmungsmaßnahmen sowie das ökonomisch unattraktivere Umfeld sank der Wanderungssaldo 2020 gegenüber dem Vorjahr um 136.078 (-35 %) ausländische Personen (vgl. Abbildung 1). Zwar pendelte sich die Zahl 2021 mit 393.342 Personen wieder auf das Vor-Corona-Niveau ein, befindet sich allerdings auf einem niedrigeren Stand als 2013. Der Anstieg war insbesondere durch erhöhte Zuwanderung aus dem asiatischen Raum (+182 % gegenüber 2020) sowie den europäischen Staaten außerhalb der EU (+43 % gegenüber 2020) getrieben. Der Wanderungssaldo von Personen aus EU-Staaten nahm hingegen seit 2015 (bis auf 2017) kontinuierlich von ehemals 327.578 auf 89.617 Personen ab. Auch für 2021 konnte kein Anstieg beobachtet werden. Am aktuellen Rand sind die Migrationsbewegungen deutlich von den aktuellen Kriegsereignissen geprägt. So registrierte Deutschland zwischen Ende Februar und dem 8. Juni 855.000 Ukrainer:innen (Brücker et al., 2022).

Aufgrund der Bedeutung der ökonomischen Perspektive für die Migrationsdynamik erscheint ein gesonderter Blick auf die aktuelle Arbeitsmarktsituation der Einwandernden von besonderem Interesse. Ins Blickfeld werden dabei Staatsangehörige von vier Ländergruppen gerückt, die einen erheblichen Anteil des Fachkräftebedarfs abdecken: Die elf Staaten der EU-Osterweiterung (EU-Ost), vier südeuropäischen Staaten (GIPS), die Staaten des Westbalkans und gesondert die Türkei.[1]

Trotz der schwierigen aktuellen Lage zeigt sich der deutsche Arbeitsmarkt bisher unbeeindruckt. Im Juni 2022 befindet sich die Zahl der Arbeitslosen stabil auf einem niedrigen Niveau, wenngleich eine erhebliche Verschlechterung der ökonomischen Erwartungen im Juli von den Unternehmen zu beobachten ist (ifo Institut, 2022). Die Ar-

1 EU-Ost: Bulgarien, Estland, Kroatien, Lettland, Litauen, Polen, Rumänien, Slowakei, Slowenien, Tschechien, Ungarn; GIPS: Griechenland, Italien, Portugal, Spanien; Westbalkan: Albanien, Bosnien und Herzegowina, Kosovo, Montenegro, Nordmazedonien, Serbien. Die Einteilung orientiert sich am Migrationsmonitor der Bundesagentur für Arbeit.

Abbildung 1
**Wanderungssaldo zwischen Deutschland und dem Ausland (ausländische Staatsbürger:innen)**
in Mio.

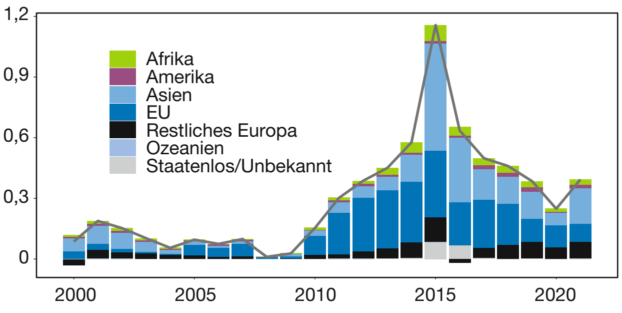

Quelle: Statistisches Bundesamt (2022); eigene Darstellung.

beitslosenquote ist im Juni 2022 gegenüber dem Vorjahreswert von 5,7 % auf 5,2 % gesunken, was einer Reduktion von 251.000 Arbeitslosen entspricht. Verglichen mit Mai 2022 ist ein leichter Anstieg von 103.000 Arbeitslosen zu verzeichnen. Dieser lässt sich teils durch einen starken Anstieg registrierter Ukrainer:innen im Zuge des Ukrainekriegs erklären. Auch der Blick auf die Zahl der Erwerbstätigen zeigt im Mai mit einem Anstieg von 1,7 % (nicht saisonbereinigt) eine positive Veränderung gegenüber dem Vorjahreswert (Bundesagentur für Arbeit, 2022a). Der akute Fachkräftemangel schlägt sich weiterhin im Stellenindex der Bundesagentur für Arbeit nieder, der trotz eines Rückgangs im Juni auf recht hohem Niveau verbleibt. Er erfasst die saisonbereinigte Arbeitsnachfrage, indem die von der Bundesagentur erfassten offenen Stellenangebote miteinbezogen werden (Bundesagentur für Arbeit, 2022b).

Wie bei den Deutschen begann eine leichte Entspannung des Arbeitsmarkts für ausländische Personen im Frühjahr 2021 und setzte sich kontinuierlich bis April bzw. Juni 2022 fort. Betrug die Arbeitslosenquote im April 2021 für Deutsche noch 5,8 % und für ausländische Staatsbürger:innen 15,3 %, so fielen diese Werte im April 2022 auf 4,8 % und 12,2 %. Verglichen mit der Zeit vor Corona 2019 ergeben sich jedoch für die Ländergruppen leichte Unterschiede mit Blick auf die arbeitsmarktliche Erholung. Gegenüber der Arbeitslosenquote im April 2019 reduzierte sich diese für Personen des Westbalkans (von 11 % auf 10,2 %), wohingegen für Personen der GIPS-Staaten (von 8,2 % auf 8,3 %), der EU-Osterweiterung (von 7,1 % auf 7,5 %) sowie der Türkei (von 14,2 % auf 15 %) die Arbeitslosenquoten leicht anstiegen.

Eine differenzierte Betrachtung der Staatengruppen nach dem Anforderungsniveau spiegelt den aktuellen Fachkräftebedarf wider. Über alle Staatengruppen hinweg absorbierte der Arbeitsmarkt erhebliche Teile der vorhandenen Fachkräfte. Im Juni 2022 reduzierte sich die Zahl der deutschen Arbeitslosen in diesem Bereich gegenüber dem Juni 2019 um 22 %, sodass die negativen Pandemieeffekte als überwunden gelten können (vgl. Abbildung 2). Eine ähnlich starke Reduktion der Arbeitslosenzahl lässt sich auch für die Gruppe der GIPS-Staaten beobachten (-21 %), gefolgt von türkischen Staatsangehörigen (-16 %). Demgegenüber fällt die Veränderung der Arbeitslosigkeit in der Gruppe des Westbalkans (-6 %) und der EU-Osterweiterung (-2 %) deutlich ab. Interessanterweise wirkten sich die konjunkturelle Dynamik und der Pandemieverlauf innerhalb der stark umworbenen Fachkräfte je nach Staatengruppe unterschiedlich stark aus. So reagierte die prozentuale Veränderung der Arbeitslosenzahlen der GIPS-Staaten während des Pandemieverlaufs deutlich volatiler als die der türkischen Staatsangehörigen.

Abbildung 2
**Prozentuale Veränderung der Arbeitslosenzahlen (Juni) für Fachkräfte gegenüber 2019**

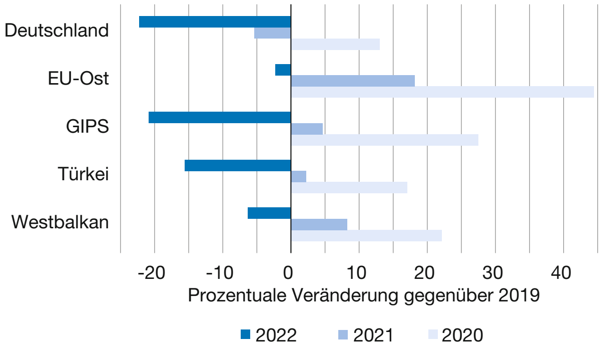

Quelle: Bundesagentur für Arbeit (2022c); eigene Darstellung.

Trotz der aktuell ökonomisch angespannten Lage erweist sich der Arbeitsmarkt als robust und absorbiert bereitwillig große Teile der ausländischen Fachkräfte. Ein besseres Verständnis der konjunkturellen Abhängigkeit der Arbeitslosigkeit einzelner Staatengruppen könnte dabei helfen, zielgerichtetere arbeitsmarktpolitische Instrumente zu entwickeln und dadurch das Fachkräfteangebot zu erhöhen. Der Ruf nach mehr ausländischen Fachkräften sollte dabei aber auch stets die Lebensumstände und Bedürfnisse der einzelnen Betroffenen mit ins Blickfeld rücken.

Erik Haustein
Helmut-Schmidt-Universität,
Hamburgisches WeltWirtschaftsInstitut (HWWI)


Literatur

Bundesagentur für Arbeit (2022a), Monatsbericht zum Arbeits- und Ausbildungsmarkt, Juni, https://statistik.arbeitsagentur.de/Statistikdaten/Detail/202206/arbeitsmarktberichte/monatsbericht-monatsbericht/monatsbericht-d-0-202206-pdf.pdf?__blob=publicationFile&v=1, (26. Juli 2022).
Bundesagentur für Arbeit (2022b), Kurzinfo: Der BA-X im Juni 2022: Nachfrage nach Arbeitskräften sinkt leicht auf hohem Niveau, https://statistik.arbeitsagentur.de/Statistikdaten/Detail/202206/arbeitsmarktberichte/bax-ba-x/ba-x-d-0-202206-pdf.pdf?__blob=publicationFile&v=3, (26. Juli 2022).
Bundesagentur für Arbeit (2022c), Statistik: Migrationsmonitor, Juni, https://statistik.arbeitsagentur.de/SiteGlobals/Forms/Suche/Einzelheftsuche_Formular.html?nn=1479694&topic_f=migrationsmonitor (26. Juli 2022).
Burstedde, A. und F. Koneberg (2022), Fachkräftemangel im Flugverkehr, IW-Kurzbericht, 52.
DIHK — Deutscher Industrie- und Handelskammertag (2022), DIHK-Vorschläge zur Reform des Fachkräfteeinwanderungsgesetzes, https://www.dihk.de/de/themen-und-positionen/fachkraefte/dihk-vorschlaege-zur-reform-des-fachkraefteeinwanderungsgesetzes-76422 (26. Juli 2022)
Ifo Institut (2022), ifo Geschäftsklimaindex deutlich gefallen, https://www.ifo.de/fakten/2022-07-25/ifo-geschaeftsklimaindex-deutlich-gefal-len-juli-2022 (26. Juli 2022).
Statistisches Bundesamt (2022), Wanderungsstatistik, https://www-genesis.destatis.de/genesis/online?operation=statistic&levelindex=0&levelid=1658859268340&code=12711#abreadcrumb (26. Juli 2022).
Tagesschau (2022), Warum das Chaos noch kein Ende nimmt, https://www.tagesschau.de/wirtschaft/unternehmen/flugchaos-verkehrsminister-heil-faeser-103.html (26. Juli 2022).
